# Sexwork and transactional sex in chemsex: results of an anonymous online study

**DOI:** 10.1192/j.eurpsy.2025.949

**Published:** 2025-08-26

**Authors:** M. Gertzen, D. Dubrovin Leao, N. Khorikian-Ghazari, T. Halms, A.-M. Strasser, A. Rabenstein, T. Rüther, A. Hasan

**Affiliations:** 1 Psychiatry and Psychotherapy, Faculty of Medicine University of Augsburg, Augsburg; 2 Psychiatry and Psychotherapy, LMU Klinikum, München, Germany

## Abstract

**Introduction:**

Sex work and transactional sex (SWTS), along with Chemsex, are linked to high-risk sexual behaviors and poorer health outcomes. Chemsex is the use of methamphetamine, GHB/GBL and mephedrone in a sexual context especially among men who have sex with men (MSM). Transactional sex (TS) is the exchange of sexual services for other services or things in a non professional way. Sexwork (SW) means having sex with people in exchange for money in a professional way.

**Objectives:**

The aim of our study was to determine the prevalence rates of sex work and transactional sex (SWTS) among chemsex users and to answer the question of whether the combination of the two leads to an increased risk profile of those affected in terms of sexual health.

**Methods:**

To achieve this, we conducted an online survey across three European German-speaking countries, targeting MSM. The survey gathered data on participants’ substance use patterns, sexual behaviors, and health outcomes, with a particular focus on their engagement in SWTS. SWTS was defined as the exchange of sexual favors for money, drugs, or other material goods, which has been shown to be associated with higher risk behaviors, including unprotected sex and multiple sexual partners.

**Results:**

A total of 399 sexually active MSM were included, categorized into three sub-groups: 129 engaging in Chemsex (MSM-CX), 128 in sexualized substance use with non-Chemsex substances (MSM-SSU), and 142 not engaging in sexualized substance use (MSM-NSU). MSM-CX reported significantly higher rates of SWTS compared to both MSM-SSU (p=.032) and MSM-NSU (p<.001), indicating that Chemsex use is strongly linked to transactional sex. Both Chemsex and SWTS were associated with higher HIV (p<.001, p=.042) and STI (p<.001, p=.023) prevalence, but no cumulative effect was found. Among MSM-CX engaging in SWTS, participants were younger (p=.006), had more sexual partners (p=.029), and reported higher substance use, including methamphetamine, mephedrone, and GHB/GBL. These factors may contribute to the increased vulnerability to HIV and STIs.

**Conclusions:**

The results highlight the need for targeted prevention and intervention measures addressing the risks of Chemsex and SWTS among MSM. Public health campaigns should consider the socio-behavioral traits of Chemsex users, such as their younger age, higher number of sexual partners, and frequent substance use. These efforts should also reduce stigma, encourage help-seeking, and promote safer sex practices. Prevention should focus on regular HIV/STI testing and accessible harm reduction strategies. In conclusion, this study underscores the importance of tailored, evidence-based interventions to improve health outcomes for MSM-CX engaging in Chemsex and SWTS.

**Disclosure of Interest:**

None Declared

